# Supplementary material for: Urine Metabolome during Parturition
Source: Metabolites. 2020 Jul 16;10(7):290. doi: 10.3390/metabo10070290 (PMC7407522; doi:10.3390/metabo10070290)
Supplement: Supplementary file 1 [file metabolites-10-00290-s001.pdf]

Supplementary Material

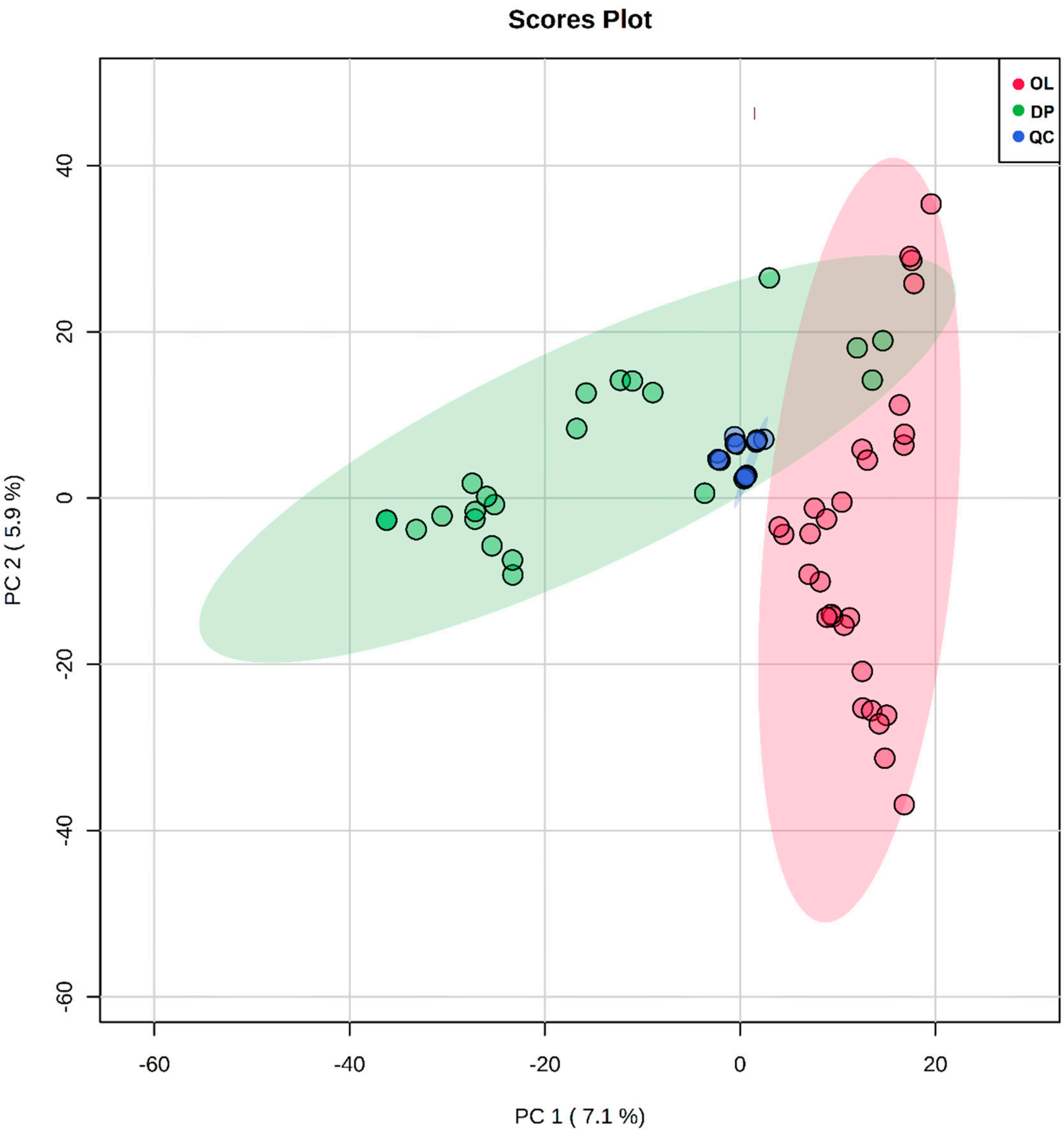

Figure S1. PCA analysis of metabolite profiling data. PCA was performed using urinary metabolomics of two sample sets OL (red) and DP (green).

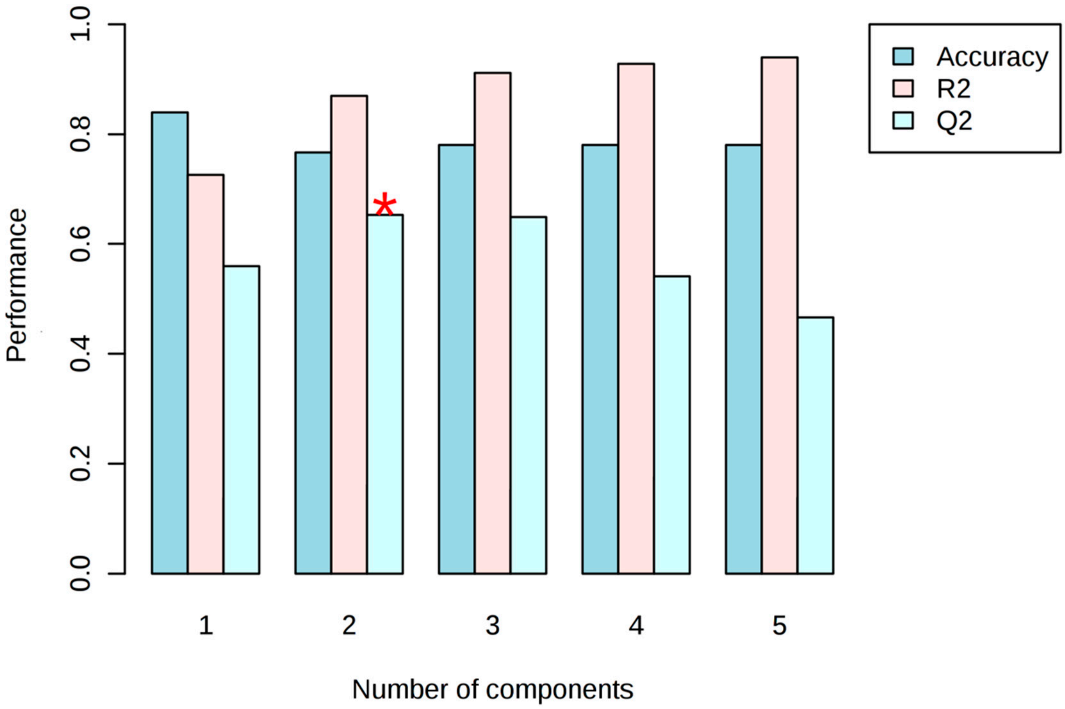

Figure S2. Cross validation chart. Blue bars: accuracy of the model; rose bars: R2 (variations); cyano: Q2 (prediction of the model). (*) Indicates a component with a higher power of projection for differentiation among the group.

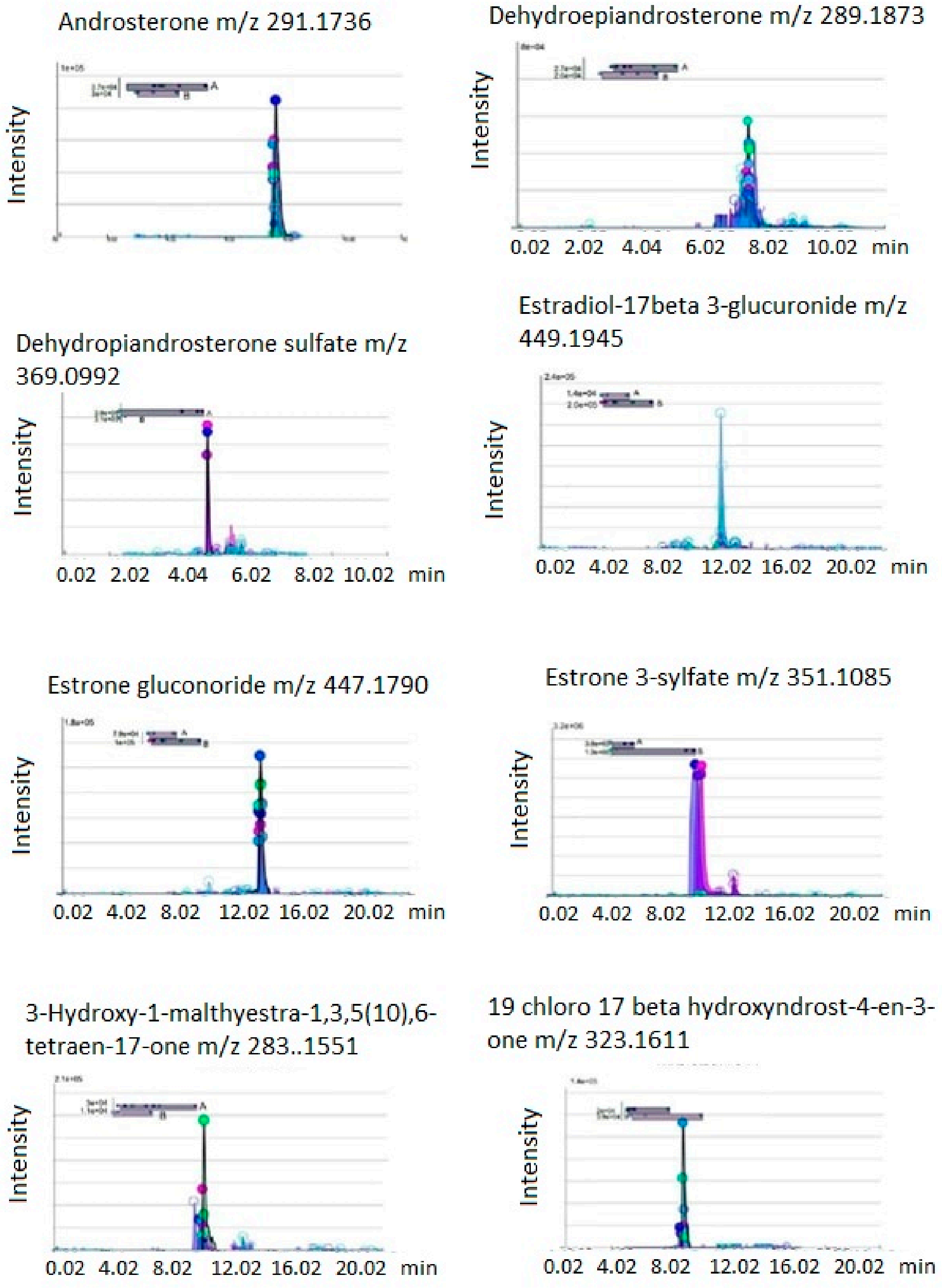

Figure S3. Electron ion chromatograms (EICs) of estrogens. (**A**) Samples collected in OL and (**B**) collected in IL-DP. The circle at the top of each EIC, represents an auto-generated quality score, with larger circles denoting higher quality of extraction. Estrogen peaks were obtained with a 5 ppm window, representing the mass accuracy of the instrument.

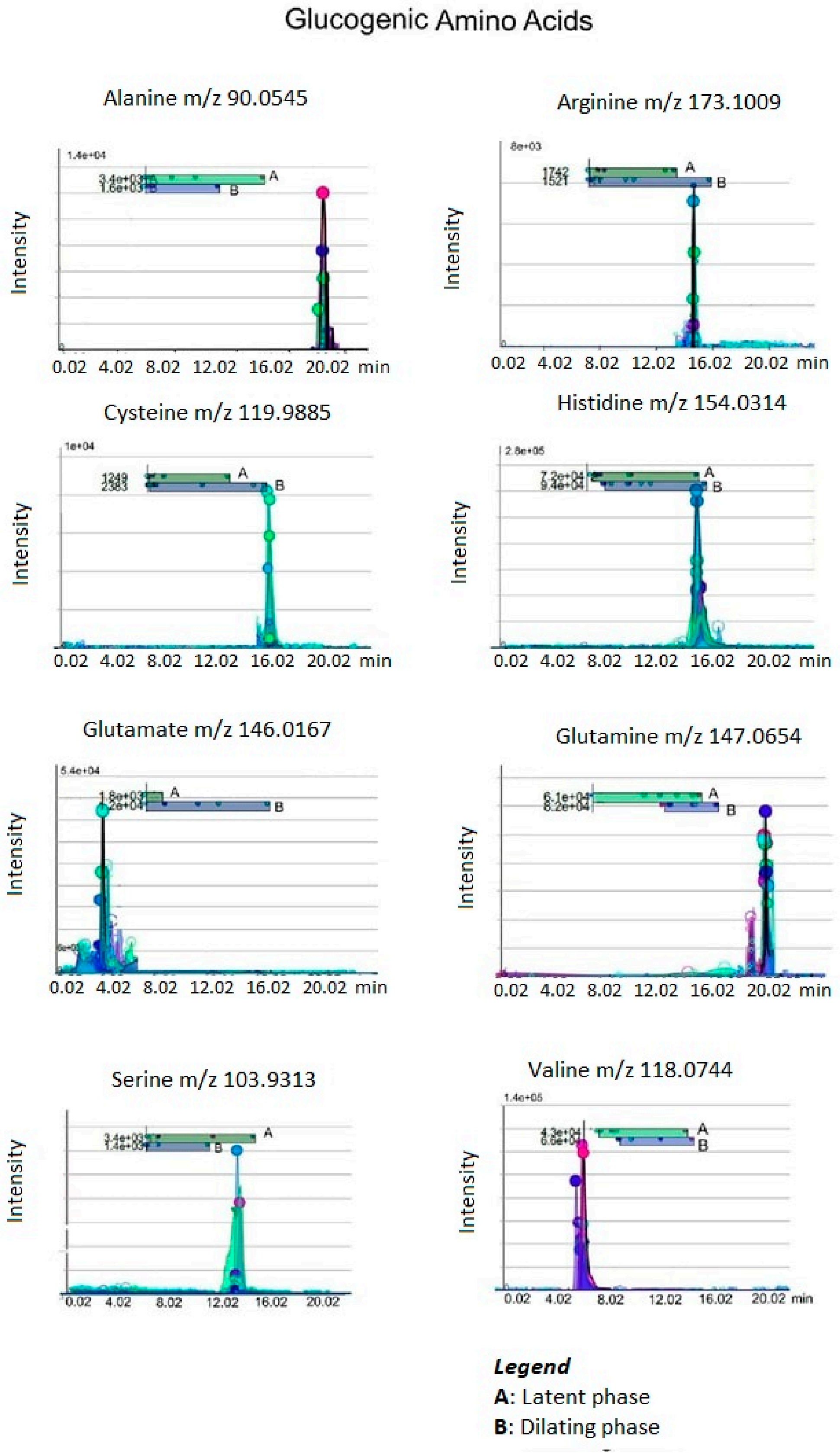

Figure S4. Electron- ion chromatograms (EICs) of glucogenic amio acids. (**A**) Samples collected in OL and (**B**) collected in DP. The circle at the top of each EIC, represents an auto-generated quality score, with larger circles denoting higher quality. Aminoacids peaks were obtained with a 5 ppm window, representing the mass accuracy of the instrument.

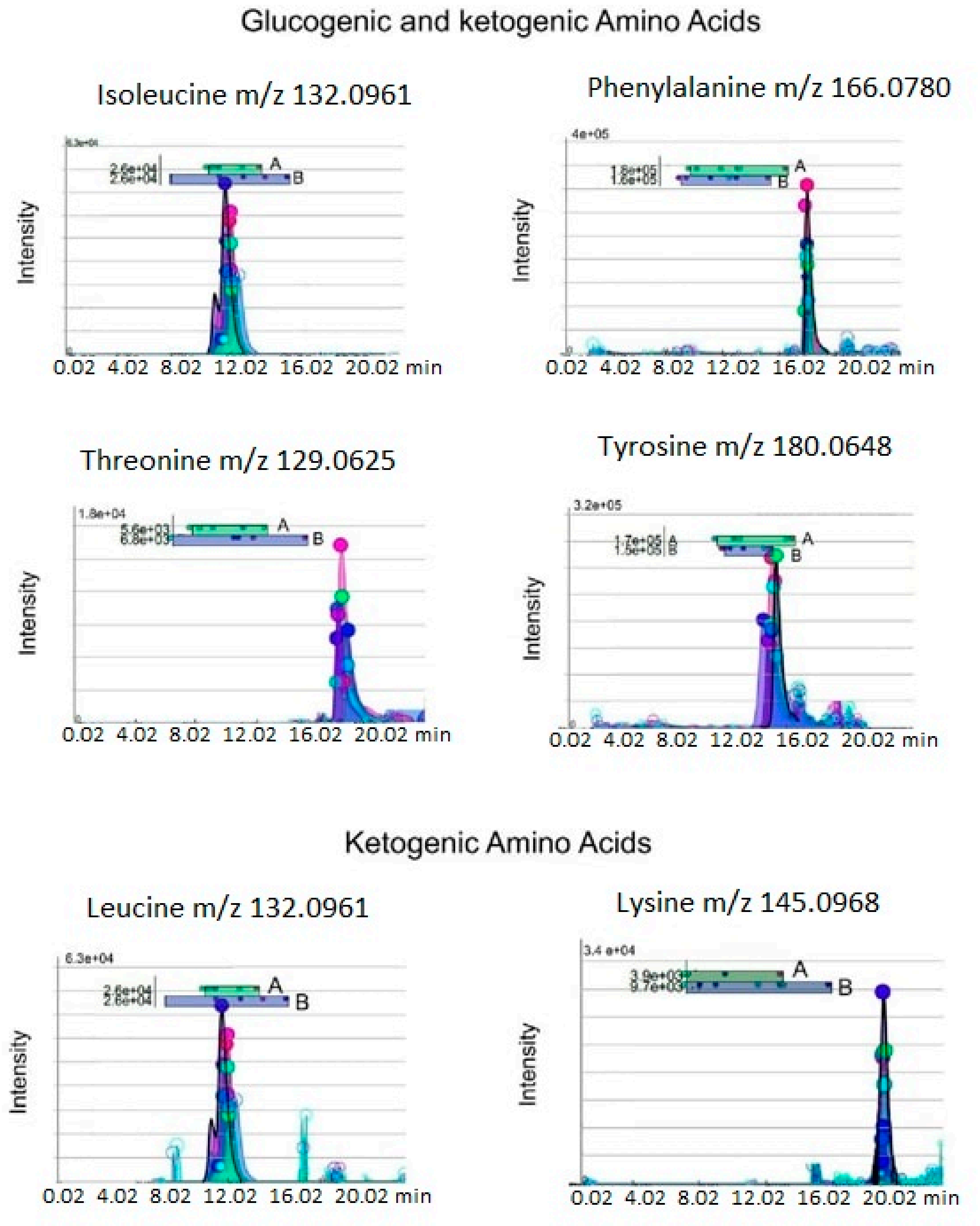

Figure S5. Electron-ion chromatograms (EICs) of ketogenic amino acids. (**A**) Samples collected in OL and (**B**) collected in DP. The circle at the top of each EIC, represents an auto-generated quality score, with larger circles denoting higher quality. Amino acids peaks were obtained with a 5 ppm window, representing the mass accuracy of the instrument.

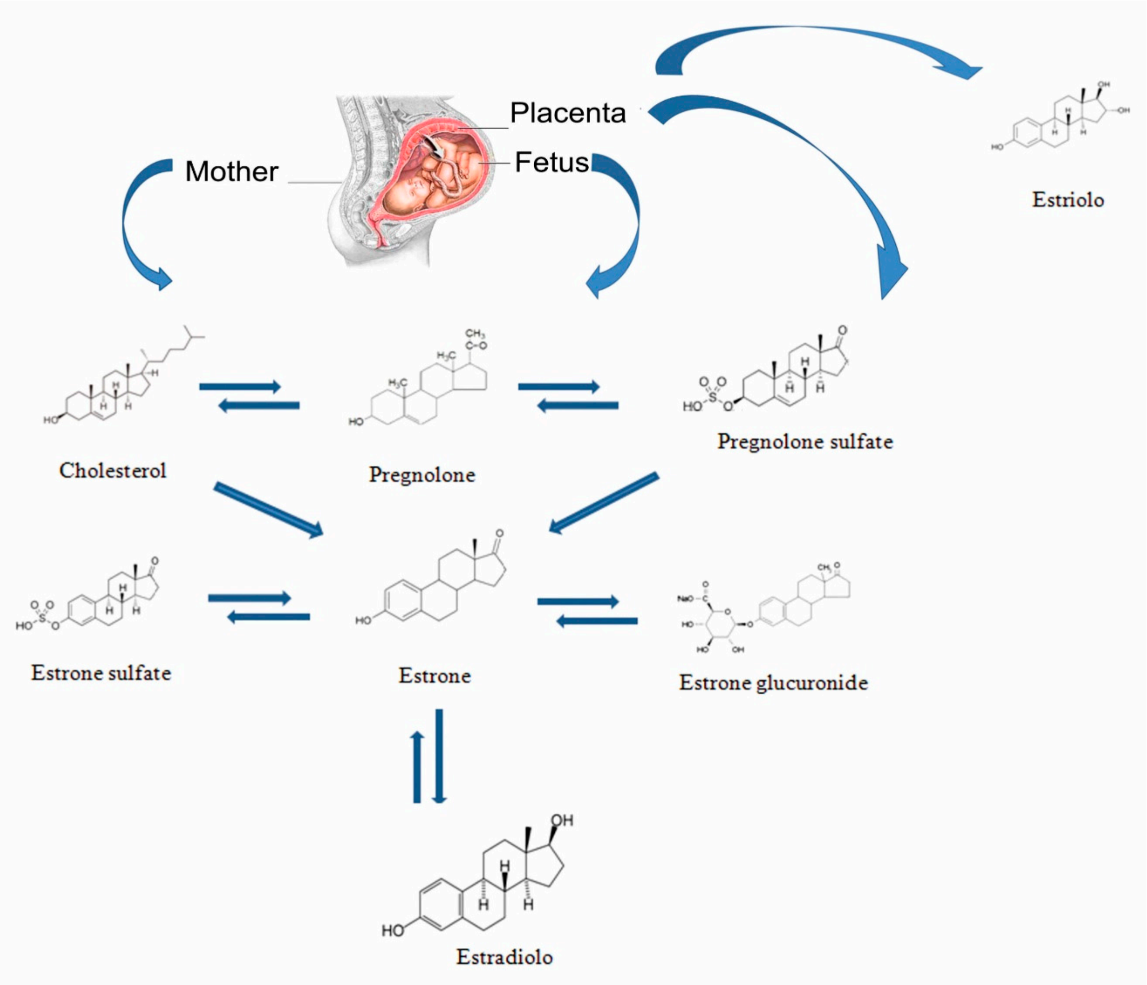

Figure S6. Scheme 1 shows the metabolism of estradiol in urine, by using our method all of these compounds could be detected simultaneously.

Table S1. The table shows the detailed results from the pathway analysis. In particular, the Total is the total number of compounds in the pathway; the Hits is the actually matched number from the user uploaded data; the Raw p is the original p value calculated from the enrichment analysis; the Holm p is the p value adjusted by Holm-Bonferroni method; the FDR p is the p value adjusted using False Discovery Rate; the Impact is the pathway impact value calculated from pathway topology analysis.

	Total Cmpd	Hits	Raw p	-log (p)	Holm adjusT	FDR	Impact
Drug metabolism - other enzymes	38	1	7.58 E-03	4.88 E+00	1.37 E-01	1.37 E-01	0.00
Glycolysis or Gluconeogenesis	31	1	1.66 E-02	4.10 E+00	2.82 E-01	1.49 E-01	0.00
Amino sugar and nucleotide sugar metabolism	88	4	4.10 E-02	3.20 E+00	6.55 E-01	1.92 E-01	0.00
Galactose metabolism	41	4	4.50 E-02	3.10 E+00	6.75 E-01	1.92 E-01	0.28
Pentose phosphate pathway	32	3	5.83 E-02	2.84 E+00	8.16 E-01	1.92 E-01	0.09
Pyrimidine metabolism	60	1	8.34 E-02	2.48 E+00	1.00 E+00	1.92 E-01	0.01
Starch and sucrose metabolism	50	4	9.04 E-02	2.40 E+00	1.00 E+00	1.92 E-01	0.02
Tyrosine metabolism	76	1	9.79 E-02	2.32 E+00	1.00 E+00	1.92 E-01	0.01
Ascorbate and aldarate metabolism	45	6	1.02 E-01	2.28 E+00	1.00 E+00	1.92 E-01	0.08
Ether lipid metabolism	23	1	1.12 E-01	2.19 E+00	1.00 E+00	1.92 E-01	0.00
Glycerolipid metabolism	32	2	1.17 E-01	2.14 E+00	1.00 E+00	1.92 E-01	0.02
Citrate cycle (TCA cycle)	20	1	1.55 E-01	1.86 E+00	1.00 E+00	2.14 E-01	0.06
Glyoxylate and dicarboxylate metabolism	50	1	1.55 E-01	1.86 E+00	1.00 E+00	2.14 E-01	0.02
Inositol phosphate metabolism	39	1	1.67 E-01	1.79 E+00	1.00 E+00	2.14 E-01	0.00
Steroid hormone biosynthesis	99	39	1.93 E-01	1.65 E+00	1.00 E+00	2.23 E-01	0.51
Pentose and glucoronate interconversions	53	7	1.99 E-01	1.62 E+00	1.00 E+00	2.23 E-01	0.43
Drug metabolism - cytochrome P450	86	1	2.64 E-01	1.33 E+00	1.00 E+00	2.79 E-01	0.00
Nicotinade and nicotinammide metabolism	44	2	5.61 E-01	5.78E-01	1.00 E+00	5.61 E-01	0.00
